# Prevalence of *Campylobacter* species in human, animal and food of animal origin and their antimicrobial susceptibility in Ethiopia: a systematic review and meta-analysis

**DOI:** 10.1186/s12941-020-00405-8

**Published:** 2020-12-10

**Authors:** Tizazu Zenebe, Niguse Zegeye, Tadesse Eguale

**Affiliations:** 1 Department of Medicine, Medical Microbiology Unit, Debre Berhan University, Debre Berhan, Ethiopia; 2 Department of Medical Laboratory Science, Debre Berhan University, Debre Berhan, Ethiopia; 3 Aklilu Lemma Institute of Pathobiology, Addis Ababa University, Addis Ababa, Ethiopia

**Keywords:** Systematic review, Meta-analysis, Prevalence, Antimicrobials Resistance, *Campylobacter* species, Ethiopia

## Abstract

**Background:**

*Campylobacter jejuni* and *Campylobacter coli* accounts for most cases of human gastrointestinal infections. The infection occurs through ingestion of contaminated food or water, and direct contact with feces of infected animal or human. Regardless of few local reports of *Campylobacter* and its antimicrobial susceptibility profile, there is no comprehensive data that show the burden of *Campylobacter* infection at national level in Ethiopia. This systemic review and meta-analysis aimed to determine the pooled prevalence of *Campylobacter* and its resistance patterns in Ethiopia from different sources.

**Method:**

A comprehensive literature search of PubMed, Google scholar, Science direct and Google engine search was conducted for studies published from 2000 to July 30, 2020 on prevalence and antimicrobial susceptibility of *Campylobacter* in human, animal and food. The study was conducted according to the Preferred Reporting Items for Systematic Reviews and Meta-Analysis (PRISMA) Checklist. Data from articles was extracted using a standardized data extraction format. The quality of the studies was assessed based on the Newcastle–Ottawa scale. The Q test and I^2^ test statistic were used to test heterogeneity across studies. The Pooled estimate of prevalence of *Campylobacter* species and its antimicrobial susceptibility profile was computed by a random effects model using STATA 16.0 software. Results were presented in forest plot, tables, funnel plot and figures with 95% confidence interval.

**Results:**

A total of 291 articles were retrieved initially. The pooled prevalence of *Campylobacter species* from different sources was 10.2% (95% CI 3.79, 16.51). In this meta-analysis, the lowest prevalence was 6.0% whereas the highest prevalence was 72.7%. In the sub-group analysis, the pooled prevalence was similar in Amhara and Oromia region, higher in Gambella and lower in Sidama. Prevalence of *Campylobacter* was higher in animals (14.6%) compared to humans (9%). The pooled antimicrobial resistance rates of *Campylobacter species* to different antimicrobials ranged from 2.9–100%. Overall, higher rate of resistance was to cephalothin (67.2%), gentamicin (67.2%), and trimethoprim-sulfamethoxazole (33.3%) in *Campylobacter* isolates from all sources. In isolates from human, resistance to cephalothin was 83% followed by amoxicillin (80%), amoxicillin-clavulnate (36%), trimethoprim-sulfamethpxazole (32%), clindamycin (31%) and ceftriaxone (28%). On the other hand, higher rate of resistance to penicillin (100%), cephalothin (60%), ciprofloxacin (71.2%), and trimethoprim-sulfamethoxazole (39%) was recorded in isolates from animals.

**Conclusion:**

The present study highlights the burden of *Campylobacter species* in the country and higher rate of resistance among investigated isolates. Designing appropriate prevention strategies and further local in-depth studies are recommended to establish actual epidemiological burden of the bacteria in the country.

## Introduction

Different enteric pathogens such as bacteria, virus, and parasites cause gastrointestinal infections in human [1]. *Campylobacter* species account for most cases of human bacterial gastrointestinal infections worldwide [2]. The genus *Campylobacter* belongs to the family Campylobacteraceae [3] and the genus *Campylobacter* contains 39 species with 16 subspecies [4]. *Campylobacter jejuni* and *C. coli* are commonly responsible to gastroenteritis in humans [3]. *Campylobacter* is one of the most frequently isolated bacteria from stools of infants with diarrhoea in developing countries, mainly due to contaminated food or water [5]. Campylobacteriosis is a common cause of human gastroenteritis in developing and industrialized countries [2]. *Campylobacter jejuni*, and *C. coli* are zoonotic pathogens where poultry, wild birds, cattle, sheep, and pigs are known source of infection [3, 6]. Poultry is a major reservoir and source of transmission of campylobacteriosis to humans [7]. Ingestion of contaminated food or water (improperly cooked poultry, untreated water, and unpasteurized milk), and direct contact with fecal material from infected animals or people are the major means of transmission of *C jejuni* and *C coli* [6]. *Campylobacter* species have well-known virulence and survival mechanisms to cause disease in human and animal [8]. In East Africa, *Campylobacter* infections have been recorded in both rural and urban areas, particularly among children and the prevalence varies between countries [9]. The spectrums of disease caused by *Campylobacter* include acute enteritis (gastroenteritis), extraintestinal infections (e.g. bacteremia, abscess, meningitis) and postinfectious complications (e.g. Guillain–Barre syndrome, reactive arthritis, and irritable bowel syndrome) [6, 7].

Most of the time, *Campylobacter* infection is a self-limiting and requires no therapeutic intervention other than supportive therapy. However, antimicrobials are employed in patients with severe, persistent, and extra intestinal campylobacteriosis and in immune-compromised patients [7]. The use of antimicrobials in agriculture led to a dramatic increase in antimicrobials resistance in several human pathogens originating from animals, including *Campylobacter* species [7].

In Ethiopia, only a few studies have been conducted on occurrence of *Campylobacter* in human, animal and food [10–14]. However, some of these studies reported high prevalence of *Campylobacter* with and reported various risk factors and antimicrobial resistance profile (13, 15). An emerging poultry production has been reported as a major contributing factor to environmental exposure to *Campylobacter* in Ethiopia (16). Plenty of reservoirs of *Campylobacter* create risk for human infection through contaminations with animal, food and environments (3, 6, 17). There is no comprehensive data that shows the burden of *Campylobacter* at national and regional level. The aim of this review was to generate comprehensive evidences on prevalence and antimicrobial susceptibility profile of *Campylobacter* in human, animal and food in Ethiopia. The findings of this study are expected to provide data that can be employed for prevention and control of *Campylobacter* infections as well as to provide gaps for further research in the country.

## Methods

### Study design

A systematic review and meta-analysis was conducted to estimate the prevalence and antimicrobials resistance patterns of *Campylobacter* in Ethiopia. The study was conducted according to the Preferred Reporting Items for Systematic Reviews and Meta-Analysis (PRISMA) Checklist [18].

### Literature search strategies for relevant studies

A comprehensive search of literatures published from 2000 to July 30, 2020 was performed in the following databases: PubMed, Google Scholar, Science Direct, Google, and manually by obtaining hard copy of locally published articles directly from authors and local libraries. *Campylobacter*, antibacterial agents, Antibiotics, Resistance, Susceptibility, and Ethiopia were used as search arms. We used search terms using Boolean operators for PubMed, Google Scholar and Science Direct: (((*Campylobacter*) AND (Antimicrobial OR Antibiotics)) AND (Resistance OR Susceptibility)) AND (Ethiopia).

### Study eligibility criteria

All available studies and data were incorporated based on the following predefined eligibility criteria. Studies conducted in Ethiopia, published articles, cross-sectional study, and articles reported in English language were used as inclusion criteria. The exclusion criteria were articles which were with duplicate or overlapping data, and articles without full text available.

### Study selection

Records identified from various sources with the search strategies were exported to Endnote reference manager software version 7. Duplicate records were identified, recorded and removed. For this, two authors independently screened the title and abstracts with the predefined inclusion criteria. Two authors were also independently assigned to collect full texts and evaluate their eligibility for final inclusion. When discrepancies between two authors occurred, the third author played a role in resolving the issue through discussion and consensus.

### Measurement of outcome variables

The outcomes of the study were prevalence of *Campylobacter* and antimicrobial resistance pattern. The prevalence was calculated by dividing the numbers of samples positive for *Campylobacter* by the total number of tested samples.

### Data extraction

All necessary data from included articles was extracted using a standardized data extraction format by two authors independently. Any disagreement during the data extraction was resolved through discussion and consensus. The primary author of the original research was contacted for additional information or to clarify method details as needed. The data extraction format for prevalence of *Campylobacter* included primary author, publication year, region, sample size, sample source, study population, diagnostic method, prevalence, species, and quality score. In addition, number of isolates, antimicrobials tested and percentage of resistance were also extracted.

### Quality assessment

Two authors independently assessed the risk of bias for each original study. The quality of study was evaluated according to Newcastle–Ottawa scale adapted for cross sectional studies [19] and graded out of 10 points (10 stars). The tool has three major sections: methodological quality (with 5 stars), comparability of the study (with 2 stars) and outcomes related to statistical analysis (with 3 stars). The mean score of two authors took for final decision, and studies with score greater than or equal to five were included for systemic review and meta-analysis (Additional file 1: Table S2).

### Data processing and analysis

The relevant extracted data was recorded using format prepared in Microsoft Excel. The data analysis was done using STATA 16.0 software. The data prepared in Microsoft excel were imported into the STATA for outcome measures and subgroup analyses. In the determination of variation in true effect sizes across population (clinical heterogeneity), restricted maximum-likehood random effect model was applied. The original articles were described using forest plot, funnel plot and tables. Random effect model was used to compute the pooled prevalence of *Campylobacter* and resistance rate. The estimated pooled prevalence with 95% confidence interval by forest plot and publication bias by funnel plot was presented. Sub group analysis was performed based on publication year, region, and study population.

Heterogeneity among reported prevalence was assessed by computing p-values of Cochrane Q-test and *I*^2^ statistics [20]. Cochrane Q-test evaluates the existence of heterogeneity and p < 0.1 was considered as statistically significant [21]. The *I*^2^ statistics provides an estimate of the percentage of the variability in effect estimates that is due to heterogeneity rather than sampling error or chance differences. I^2^ values of 25%, 50% and 75% are considered to represent low, medium and high heterogeneity respectively [20, 21]. Begg’s rank test and Egger’s regression test are among various statistical tests used for checking publication bias in the funnel plot [22]. Begg’s rank test was used to examine the correlation between the effect sizes and their corresponding sampling variances and a strong correlation implies publication bias. Egger’s test regresses the standardized effect sizes on their precisions; in the absence of publication bias, the regression intercept is expected to be zero [22]. Begg’s rank test and Egger’s tests at 5% significant level were used to check publication bias [22, 23].

## Results

**Fig. 1:**
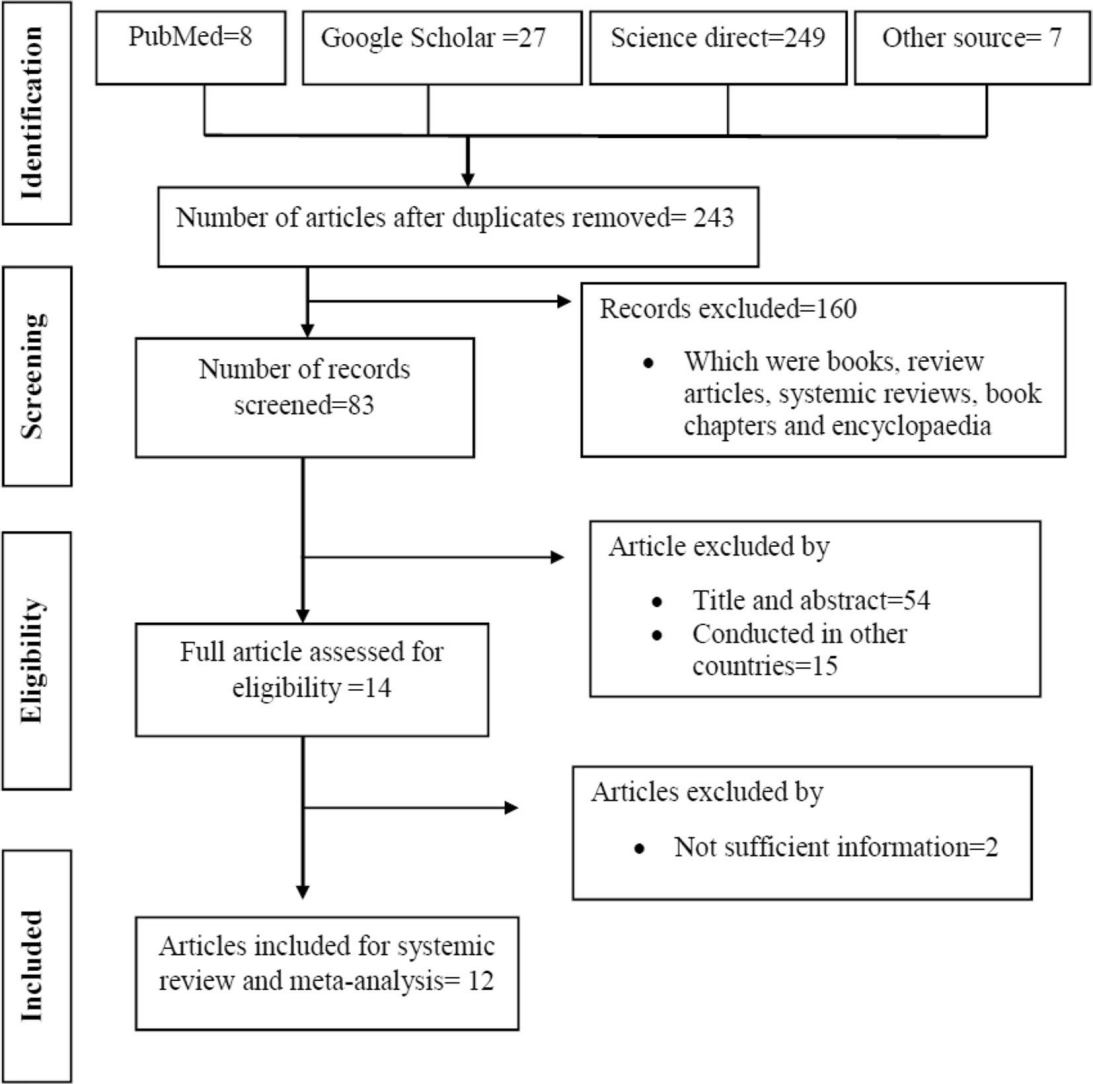
Flow chart of study selection for systematic review and meta-analysis of the prevalence of Campylobacter species in different parts of Ethiopia

A total of 291 articles were initially retrieved for the prevalence of *Campylobacter* species and antimicrobial susceptibility from different sources in Ethiopia using different data bases described above. As a result of duplication, 48 articles were removed. After further screening, 160 articles which were books, review articles, systemic reviews, book chapters and encyclopaedia were excluded. Based on the eligibility criteria, 69 articles were excluded and 14 were selected for their full text articles. Finally, 2 articles were excluded due to insufficient information and the remaining 12 articles were included for systemic review and meta-analysis (Fig. 1).

### Description of search results

All the included 12 articles (Table 1) were cross sectional in their study design and published from 2004 to 2020. The total sample size for these selected studies was 4230 (ranging from 158–540. These studies were conducted in four regional states, namely Amhara, Oromia, Sidama, and Gambella regions. Majority of the studies were conducted in Oromia [10, 15, 24–26] and Amhara [11, 13, 27, 28] region. The studies involved humans, animals and food of animal origin; more than 90% were conducted in humans and animals. Among all the studies, one study isolated *Campylobacter* from both humans and animals which were separately analysed. Data on susceptibility profile of *Campylobacter species* was included for a total of 14 antimicrobials. The inclusion of antimicrobials in each study ranged from 4-14 antimicrobials, 50% of the studies tested susceptibility of *Campylobacter* to 9 or more antimicrobials.

**Table 1:** Summary of 12 studies reporting the prevalence of *Campylobacter species* in different parts of Ethiopia, from 2004 to 2020

Study identification	Quality score	Publication year	Region	Study area	Study population	Sample size	Types of sample	Diagnosis method	Prevalence (%)			Species	
									Characteristics	Specific	Total	*C. jejuni*	*C. coli*
Beyene et al. [24]	6	2004	Oromia	Jimma	Human	430	Stool	Culture	Children	11.6	11.6	–	–
Kassa et al. [25]	7	2007	Oromia	Jimma	Animal	485	Faeces	Culture	Chicken	68.1%	39.6	70.3	26.6
									Pigs	50%			
									Sheep	38%			
									Cattle	12.6%			
Dadi et al. [10]	5	2008	Oromia	DebreZeit/Addis Ababa	Food of animal origin	540	Raw meat	Culture	Raw meat	9.3^*^	9.3^*^	78	18
Woldemariam et al. [26]	5	2009	Oromia	DebreZeit	Animal	398	Carcass	Culture	Sheep	10.6	10.1	72.5	27.5
									Goats	9.4			
Ewnetu et al. [11]	6	2010	Amhara	Bahir Dar	Human	164	Stool	Culture	Patients	8	8	94.1	5.9
					Animal	220	Cloacal samples	Culture	Chickens	72.7	72.7	92.5	7.5
Chanyalew et al. [27]	5	2013	Amhara	DebreBerhan	Animal	380	Carcass and fecal	Culture	Sheep	12.6	12.6	–	–
Lengerh et al. [13]	6	2013	Amhara	Gondar	Human	285	Stool	Culture	Under-five children	15.4	15.4	90.9	9.1
Getamesay et al. [30]	5	2014	Sidama	Hawasa	Human	158	Stool	Culture	Under-five children	12.7	12.7	–	–
Tafa et al. [15]	5	2014	Oromia	Jimma	Human	227	Stool	Culture	Under-five children	16.7^*^	16.7^*^	71.1	21.1
Abamecha et al. [31]	5	2015	Gambella	Lare	Animal	368	Faeces	Culture	Chicken	86.6%	56.5*	83.7	12.9
									Cattle	48%			
									Sheep	39%			
									Goat	33.3%			
Nigatu et al. [28]	7	2015	Amhara	Gondar	Animal	360	Faeces	Culture	Poultry	28.9%	25	56.8	43.2
									Cattle	21.5%			
Kebede et al. [29]	8	2017	Sidama	Hawasa	Human	215	Stool	Culture	HIV-patients	6.04	6.04	–	–

^a^The remaining species were C. lari

### Prevalence of *Campylobacter species*

**Fig. 2:**
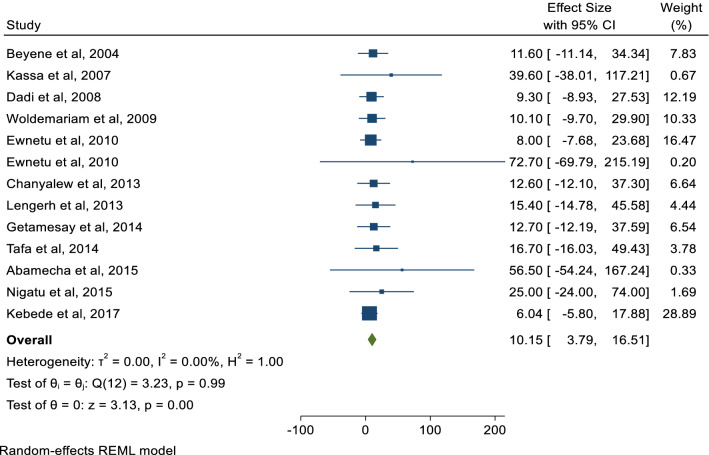
Forest plot of the pooled prevalence of Campylobacter species in different parts of Ethiopia

**Fig. 3:**
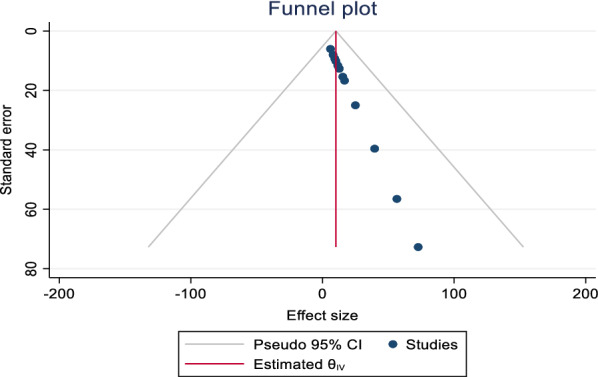
Funnel plot with 95% confidence limits of the prevalence of Campylobacter species in different parts of Ethiopia

In the present systemic and meta-analysis of 12 included articles, the pooled prevalence of *Campylobacter species* was 10.2% (95% CI 3.79, 16.51). There was no heterogeneity observed across the included studies (I^2^ 0.01%; Q = 3.23, p = 1.00). However, random effect model with restricted maximum-likelihood method was used to estimate the pooled prevalence of *Campylobacter species* from different sources in Ethiopia (Fig. 2). In this meta-analysis, the lowest prevalence was 6.1% [29] from stool sample of HIV infected individuals and highest prevalence was 72.7% from cloacal samples of chicken [11]. The *Campylobacter* species including *C. jejuni,* and *C. coli (C. lari* in few studies too) were reported at species level in 75% of the studies. Significantly predominant species with pooled prevalence of 75.0% (95% CI 25.36, 124.66) were *C. Jejuni* compared to *C. coli* 9.4% (95%CI 2.19, 16.68) in Ethiopia. Funnel plot was used to show the distribution of the studies and showed symmetrical distribution of effect estimate (Fig. 3). The Egger’s test showed that there was no statistically significant publication bias in estimating the prevalence of *Campylobacter species* from different sources (p = 0.0725).

### Subgroup analysis

A subgroup analysis for publication year, region, and study population (human, animal and food of animal origin) was done (Table 2). In most cases, sub group analysis was done to investigate source of heterogeneity (effect difference in relevant subgroups), and to provide effect estimate (prevalence) for clinically relevant subgroups [32]. Regardless of absence of heterogeneity, we performed subgroup analysis to provide effect estimate for clinically relevant subgroups. This was with assumption that the effect estimate between clinically relevant subgroups will vary. In considering the small studies effect, Trim and Fill analysis was used to adjust the final pooled estimate for each subgroup. The estimate pooled prevalence of *Campylobacter species* from different sources was not changed within the two decades period, from 2000–2010 (10.1, 95% CI 0.88, 19.31) and 2011–2020 (10.2, 95%CI 1.40, 19.00). The pooled prevalence in the two regions, Amhara (11.6, 95% CI − 0.16, 23.29) and Oromia (11.4, 95%CI 0.66, 22.23) was almost the same. Lowest prevalence was seen in Sidama region (7.3, 95%CI − 3.27, 17.96) whereas highest prevalence was seen in Gambella (56.5, 95%CI − 54.24, 167.24). Higher prevalence was seen in animals (14.6: 95%CI 0.32, 28.88) compared to human and food. The prevalence was almost similar in human and food of animal origin.

**Table 2:** Subgroup estimate pooled prevalence of *Campylobacter* species in animal, human and food in Ethiopia

Variables	Characteristics	Included studies	Sample size	Pooled prevalence (95% CI)
Publication year	2000–2010	6	2237	10.09 (0.88, 19.31)
	2011–2020	7	1993	10.20 (1.40, 19.00)
Region	Amhara	5	1409	11.57 (− 0.16, 23.29)
	Oromia	5	2080	11.44 (0.66, 22.23)
	Sidama	2	373	7.27 (− 3.27, 17.96)
	Gambella	1	368	56.50 (− 54.24, 167.24)
Study population	Animal	6	2211	14.60 (0.32, 28.88)
	Human	6	1479	9.00 (1.28, 16.72)
	Food of animal origin	1	540	9.30 (− 8.93, 27.53)

### Antimicrobial susceptibility profile of *Campylobacter* species

The percentage of antimicrobial resistance rates of *Campylobacter species* to different antimicrobials varied from 0–100% (Table 3). Over 60% of *Campylobacter* isolates were resistant to Penicillin, Ampicillin, Amoxicillin, Tetracycline, Cephalothin, Gentamicin, Ciprofloxacin, and Trimethoprim-sulfamethoxazole. Relatively, Streptomycin, Nalidixic acid, Chloramphenicol and Norfloxacin were effective to about 50% of *Campylobacter* isolates.

**Table 3:** Percentage of antibacterial resistance rates of *Campylobacter species* in Ethiopia, from 2004 to 2020

Studies	Number of isolates		Antimicrobials resistance rate reported by 12 studies (%)																
		P	AMP	AML	E	STR	T	NA	CF	GM	CIP	CL	A/C	DC	NOR	CRO	T/S	CD	K
Lengerh et al. [13]	44	–	68.2	–	27.7	–	56.8	9.1	88.9	18.2	16	11.4	36.4	15.9	11.6	27.7	54.5	40.9	–
Beyene et al. [21]	50	–	50	–	10	–	14	–	–	0	–	0	–	–	–	–	60	–	0
Dadi et al. [10]	50	–	10	6	2	20	10	–	–	14	–	4	–	–	–	–	–	–	12
Kebede et al. [27]	13	–	–	–	23.1	–	23.1	7.7	–	0	7.7	53.8	–	–	0	0	61.5	–	–
Getamesay et al. [28]	20	–	30	80	55	–	15	20	70	70	10	0	–	–	–	0	20	–	–
Chanyalew et al. [25]	48	–	33.3	–	–	4.2	20.8	2.1	100	–	–	–	–	–	–	–	–	–	–
Tafa et al. [24]	38	–	76	–	18.4	–	39.5	10.5	100	13.2	15.8	31.6	–	23.7	10.5	–	68.4	26.3	–
Ewnetu et al. [11]	15	–	18.8	–	18.8	25	22.2	12.5	–	–	–	–	–	–	–	–	–	–	–
	160	–	34.1	–	14.9	20.5	30.1	–	–	–	–	–	–	–	–	–	–	–	–
Kassa et al. [22]	192	–	19.8	–	2.6	6.3	2.6	6.3	96.9	0.5	–	–	–	–	2.2	–	38.5	2.1	–
Abamecha et al. [29]	208	–	38.9	–	50.5	–	69.2	3.8	100	–	71.2	51.4	–	–	54.3	–	–	45.7	–
Nigatu et al. [26]	84	100	8.3	–	60	13.1	35.7	0	40.5	–	–	–	–	–	–	–	–	–	–

*AMP* Ampicillin, *AML* Amoxicillin, *E* Erythromycin, *STR* Streptomycin, *T* Tetracycline, *NA* Nalidixic acid, *P* Penicillin, *CF* Cephalothin, *GM* Gentamicin, *CIP* Ciprofloxacin, *CL* Chloramphenicol, *A/C*Amoxicillin with clavulanic acid, *DC* Doxycycline, *NOR* Norfloxacin, *CRO* Ceftriaxone, *T/S* Trimethoprim-sulfamethoxazole, *CD* Clindamycin, *K* Kanamycin

The pooled resistance rates of *Campylobacter* isolates to14 antimicrobials was found to be varied, ranged from 2.9–100% (Additional file 1: Table S1). A single study showed 100% resistance of *Campylobacter* isolates to penicillin. *Campylobacter* isolates showed significantly higher resistance rate to Cephalothin (67.2%), Gentamicin (67.2%), and Trimethoprim-sulfamethoxazole (33.3%). Isolates were relatively more sensitive to Amoxicillin, Erythromycin, Streptomycin, Nalidixic acid, Chloramphenicol, and Norfloxacin but not statistically significant (p < 0.05).

A sub group analysis was performed to examine estimate pooled antimicrobial resistance rate to *Campylobacter* in human, animal and food (Table 4). The *Campylobacter* isolates from human showed higher resistance against amoxicillin (80%), cephalothin (83%), ceftriaxone (28%), amoxicillin-clavulnate (36%), trimethoprim-sulfamethpxazole (32%), and clindamycin (31%). *Campylobacter* isolates from animal also showed higher resistance to penicillin (100%), cephalothin (60%), ciprofloxacin (71.2%), and trimethoprim-sulfamethoxazole (39%). In both cases the resistance was not statistically significant. *Campylobacter* isolates from animal and human showed higher resistance to cephalothin and trimethoprim-sulfamethoxazole. The *Campylobacter* strains isolated from human showed significant resistance to erythromycin (16%) and tetracycline (19%).

**Table 4:** Percentage of pooled resistance rates of antimicrobials to *Campylobacter* isolates from human, animal and food in Ethiopia, from 2004 to 2020

Antimicrobials	Pooled antimicrobial resistance rate (95% CI)		
	Human	Animal	Food
Penicillin	–	100 (− 96, 296)	–
Ampicillin	28.41 (− 0.09, 56.91)	13.14 (− 0.90, 27.18)	10 (− 9.6,, 29.6)
Amoxicillin	80 (− 76.8, 236.8)	–	6 (− 5.76, 17.76)
Erythromycin	15.82 (1.7, 29.93)	4.01 (− 3.86, 11.89)	2 (− 1.92, 5.92)
Streptomycin	25 (− 24, 74)	5.79 (− 0.74, 12.32)	20 (− 19.2, 59.2)
Tetracycline	18.7 (2.37, 35.04)	10.37 (− 7.03, 27.77)	10 (− 9.6, 29.6)
Doxycycline	18.32 (− 7.56, 44.2)	–	–
Nalidixic acid	9.91 (0.86, 18.96)	2.8 (− 0.66, 6.25)	–
Cephalothin	82.52 (− 11.93, 176.97)	60.05 (− 4.71, 124.8)	–
Ceftriaxone	27.7 (− 26.59, 81.99)		–
Gentamicin	16.18 (− 4.53, 36.88)	0.5 (− 0.48, 1.48)	14 (− 13.44, 41.44)
Ciprofloxacin	10.23 (− 0.23, 20.74)	71.2 (− 68.35, 210.75)	–
Chloramphenicol	15.26 (− 5.35, 35.87)	51.4 (− 49.34, 152.14)	4 (− 3.84, 11.84)
Amoxicillin-clavulanate	36.4 (− 34.94, 107.74)	–	–
Norfloxacin	11 (− 4.26, 26.25)	2.29 (− 2.02, 6.59)	–
Trimethoprim-sulfamethoxazole	32.26 (− 0.44, 64.96)	38.5 (− 36.96, 113.96)	–
Clindamycin	30.57 (− 12.79, 73.93)	2.19 (− 1.92, 6.30)	–
Kanamycin	–	–	12 (− 11.52, 35.52)

## Discussion

Human campylobacteriosis is a public health concern globally because of rise in the incidence of *Campylobacter* infection, and trend of increasing resistance to antimicrobial agents [33]. Thermotolerant *Campylobacter* species *(C jejuni, C coli, C. lari*, and *C. upsaliensis)* have clinical relevance in human [6]. Currently, the most relevant species within the genus is *C. jejuni*, a leading cause of bacterial gastroenteritis in humans followed by *C. coli* (1 to 25% of all *Campylobacter*-related diarrheal diseases) [34]. *Campylobacter jejuni* is also the most significant pathogenic *Campylobacter* in animals followed with other species such as *C. fetus* [35]. However, today there are other emerging *Campylobacter* species that cause disease in human and animals [34]. Most of the studies done in Ethiopia included only *C. jejuni,* and *C. coli*. Majority of studies used in the present systematic review and meta-analysis reported only C. *jejuni* and *C. coli* in different sources (human, animal and food).

The pooled prevalence of *Campylobacter species* in the present systemic review and meta-analysis was 10.2% with *C. jejuni* as predominant species. Systemic review conducted on studies conducted in Sub-Saharan Africa showed that prevalence of *Campylobacter species* ranged from 1.7%–62.7% in humans and 1.2%–80% in animals [36]. Ethiopia as part of Sub-Saharan Africa country shares the burden of *Campylobacter* infection. In the sub-group analysis, during the two decades, there was no change in prevalence of *Campylobacter*. This is presumably due to the fact that there was no specific intervention strategy conducted to prevent and control *Campylobacter.* Studies from other parts of the world showed increasing incidence and prevalence of *Campylobacter species* as emerging infections [34] which need serious attention against burden of *Campylobacter* infection.

The pooled prevalence in Amhara region (11.6%) and Oromia (11.4%) was almost the same. This similarity may be due to communality of contributing factors such as day to day activities, agricultural practice, and life style of the communities who live in these regions which could have contributed to the transmission of *Campylobacter* infection in similar manner. However, one of the studies conducted in Amhara region reported relatively higher prevalence of *Campylobacter* (72.7%) compared to the other reports in both regions. This may be due to difference in study population, for example this study was conducted in chickens. Chickens appear to be important hosts for *Campylobacter* compared to the other species [4] and suggests the importance of chicken in the transmission of *Campylobacter* in the area. In majority of the studies included in the present systemic review and meta-analysis, higher prevalence of *Campylobacter* was reported from Chicken. Lower prevalence was reported in study from Sidama region (7.3%) whereas higher prevalence was reported from Gambella (56.5%). This may be due to few numbers of studies done in these regions (report bias) or actual difference. Hence, there is a need for more research in the area.

Higher prevalence was reported in animals (14.6%) compared to human and food. And this may be due to the fact that most of domestic animals can serve as reservoir for *Campylobacter* [4]. A systematic review and meta-analysis of *Campylobacter* from food animals and meat in Africa reported 37.7% prevalence estimate of *Campylobacter* [37]. Gahamanyi et al. [36] also reported prevalence of *Campylobacter* in animal ranged from 1.2%–80%. The higher prevalence in the present study therefore agreed with studies conducted elsewhere and could suggest the importance of *Campylobacter* related to animals in Ethiopia. In the sub-group analysis, the pooled prevalence of *Campylobacter species* in human was 9% which is lower compared to reports by Gahamanyi et al. [36], 62.7% in Nigeria, 21% in Malawi, and 20.3% in South Africa. The difference may be due to difference in detection methods used by different studies. A recent study done in Eastern Ethiopia reported 50% using PCR and 88% using meta-total RNA sequencing from stool of children [38]. The mean prevalence of *Campylobacter* in Sub-Saharan Africa was 18.6% for all age groups and 9.4% for under-five children [36]. This is in line with the present estimation, particularly for under-five children. The study population in majority of the present study for human were children or under-five children (66.7%). This suggests that the burden of *Campylobacter* in under-five children in Ethiopia is similar to other Sub-Saharan Africa countries. *Campylobacter* is well recognized as the leading cause of bacterial foodborne diarrheal diseases [39]. The 9.3% prevalence of *Campylobacter* in a single study in raw meat agrees with Pallavi et al. [40] who reported 17.3% in chicken meat.

Resistance rates of *Campylobacter species* to different antimicrobials varied from 0-100% in the present study. This agrees with Gahamanyi et al. [36] who reported resistance to commonly used antimicrobials ranging from 0–100%. The pooled rate of resistance of *Campylobacter* isolates to different antimicrobials was erythromycin (3.1%), ampicillin (14.3%), tetracycline (17.1%), nalidixic acid (3.7%), and ciprofloxacin (10.6%) in the present study. Contrary to this, Hlashwayo et al. [4] reported higher resistance rate to erythromycin (44%), ampicillin (39%), tetracycline (33%), nalidixic acid (31%) and ciprofloxacin (30%). This discrepancy may be due to difference in study population, the present study include human, animal and food whereas the previous study was based only on animal isolates. In the present study *Campylobacter* species showed significantly higher resistance rate to cephalothin (67.2%), gentamicin (67.2%), and trimethoprim-sulfamethoxazole (33.3%). Trends in fluoroquinolone-resistance in samples from animal and human showed increased resistance among isolates of members of the genus *Campylobacter* [41]. Importantly, based on the present systemic and meta-analysis, antimicrobials with good activity against *Campylobacter* species included amoxicillin, erythromycin, streptomycin, nalidixic acid, chloramphenicol, and norfloxacin.

The present finding highlights the frequent occurrence of resistance strains of *Campylobacter* to commonly used antimicrobials in human, animal and food in Ethiopia (Table 4). The *Campylobacter* isolated from human and animal showed higher resistance against commonly used antimicrobials. The finding agrees with Hlashwayo et al. [4] reports in which most *Campylobacter* isolates were resistant to erythromycin (44%), ampicillin (39%), tetracycline (33%), nalidixic acid (31%) and ciprofloxacin (30%). The *Campylobacter* isolated from human showed significant resistance against erythromycin (16%) and tetracycline (19%). Antimicrobial use in agricultural industry considered as a root cause of antimicrobials resistance in *Campylobacter* and other foodborne pathogens [42]. In addition misuse of antimicrobials in poultry industry is found to be source for antimicrobial resistant bacterial isolates in Ethiopia [43]. This could be due to irrational use of antimicrobials in food production in Ethiopia [44].

Majority of the studies were done by culture only which is a challenge for the growth of *Campylobacter* for reporting actual prevalence. The other limitations include less number of studies done in Ethiopia, absence of data from other regions and few number of articles were included for this systemic and meta-analysis. Few studies were available in food, animals and human that limited comparisons of the prevalence and resistance profile.

## Conclusion

The pooled prevalence of *Campylobacter species* was 10.2% with higher prevalence in animal and predominant species was *C. jejuni.* The prevalence of *Campylobacter species* varied among region. The study found different levels of occurrence of resistant *Campylobacter* strains in human, animal and food in Ethiopia. *Campylobacter* isolated either from human or animal or foods were showed higher resistance against amoxicillin, cephalothin, ceftriaxone, amoxicillin-clavulnate, clindamycin, penicillin, ciprofloxacin, and trimethoprim-sulfamethoxazole. Majority of the studies were done in Amhara and Oromia region, and the design did not include other emerging species.

The present study highlights the burden of *Campylobacter species* and showed gap of data on *Campylobacter* in many other regions of the country. Infection with resistant strains of *Campylobacter* could challenge the management of the infection unless the right prevention strategies are design. Further studies involving advanced techniques and targeting other emerging *Campylobacter species* are recommended to understand full picture of the burden *Campylobacter* in the country. Furthermore, studies focusing on the transmission dynamics of resistance strains of *Campylobacter* between human, animal and food is recommended.

## Supplementary information

**Additional file 1: Table S1.** Percentage of pooled antimicrobial resistance rates of 14 antimicrobials to *Campylobacter* isolates in Ethiopia, from 2004 to 2020. **Table S2.** Critical appraisal of studies.

## Data Availability

There is no remaining data and materials; all information is clearly presented in the main manuscript.
