# Supplementary material for: Prevalence of Campylobacter species in human, animal and food of animal origin and their antimicrobial susceptibility in Ethiopia: a systematic review and meta-analysis
Source: Ann Clin Microbiol Antimicrob. 2020 Dec 10;19:61. doi: 10.1186/s12941-020-00405-8 (PMC7731538; doi:10.1186/s12941-020-00405-8)
Supplement: Supplementary file 1 — Additional file 1: Table S1. Percentage of pooled antimicrobial resistance rates of 14 antimicrobials to Campylobacter isolates in Ethiopia, from 2004 to 2020. Table S2. Critical appraisal of studies. [file 12941_2020_405_MOESM1_ESM.docx]

**Table S1.** Percentage of pooled antimicrobial resistance rates of 14 antimicrobials to *Campylobacter* isolates in Ethiopia, from 2004 to 2020

| Antimicrobials | Included studies | Number of isolates | Pooled resistance rate (95% CI) |
| --- | --- | --- | --- |
| Penicillin | 1 | 84 | 100 (-96, 296) |
| Ampicillin | 11 | 909 | **14.33 (3.74, 24.93)** |
| Amoxicillin | 2 | 86 | 6.41 (-5.31, 18.14) |
| Erythromycin | 11 | 874 | 3.06 (0.05, 6.08) |
| Streptomycin | 6 | 519 | 6.50 (0.11, 12.88) |
| Tetracycline | 12 | 922 | **17.12 (5.57, 28.66)** |
| Nalidixic acid | 9 | 662 | 3.70 (0.47, 6.93) |
| Cephalothin | 7 | 634 | **67.23 (13.82, 120.64)** |
| Gentamicin | 7 | 407 | **67.23 (13.82, 120.64)** |
| Ciprofloxacin | 5 | 323 | 10.57 (0.09, 21.05) |
| Chloramphenicol | 7 | 423 | 5.66 (-1.64, 12.97) |
| Amoxicillin-clavulanate | 1 | 44 | 36.40 (-34.94, 107.74) |
| Doxycycline | 2 | 82 | 18.32 (-7.56, 44.20) |
| Norfloxacin | 5 | 495 | 2.93 (-1.23, 7.10) |
| Ceftriaxone | 2 | 77 | 18.32 (-7.56, 44.20) |
| Trimethoprim-sulfamethoxazole ( | 6 | 357 | **33.25 (3.24, 63.25)** |
| Clindamycin | 4 | 482 | 10.18 (-10.96, 31.32) |
| Kanamycin | 2 | 100 | 12.00 (-11.52, 35.52) |

**Table S2. Critical appraisal of studies**

| Studies | Methodological quality (with 5 stars) | | | | Comparability of the study (with 2 stars) | outcomes with related to statistical analysis (with three stars) | | Total, of 10% |
| --- | --- | --- | --- | --- | --- | --- | --- | --- |
|  | Representativeness of the sample(1) | Sample size (1) | Non-respondents (1) | Ascertainment of the exposure (risk factor) (1 or 2) | The subjects in different outcome groups are comparable, based on the study design or analysis (1 or 2). | Assessment of the outcome (1 or 2) | Statistical test (1) |  |
| Chanyalew *et al,* 2016 | 0 | 0 | 0 | 1 | 1 | 1 | 1 | 4 |
| Lengerh *et al,* 2013 | 1 | 1 | 0 | 1 | 1 | 1 | 1 | 6 |
| Beyene *et al,* 2004 | 1 | 1 | 0 | 1 | 1 | 1 | 1 | 6 |
| Dadi *et al,* 2008 | 1 | 1 | 0 | 0 | 1 | 1 | 1 | 5 |
| Kebede *et al,* 2017 | 1 | 1 | 1 | 1 | 2 | 1 | 1 | 8 |
| Woldemariam *et al,* 2009 | 1 | 1 | 0 | 0 | 1 | 1 | 1 | 5 |
| Getamesay *et al,* 2014 | 1 | 1 | 0 | 0 | 1 | 1 | 1 | 5 |
| Chanyalew *et al,* 2013 | 1 | 1 | 0 | 0 | 1 | 1 | 1 | 5 |
| Tafa *et al,* 2014 | 1 | 1 | 0 | 0 | 1 | 1 | 1 | 5 |
| Ewnetu *et al,* 2010 | 1 | 1 | 0 | 1 | 1 | 1 | 1 | 6 |
| Kassa *et al,* 2007 | 1 | 1 | 0 | 1 | 1 | 2 | 1 | 7 |
| Abamecha *et al,* 2015 | 1 | 1 | 0 | 0 | 1 | 1 | 1 | 5 |
| Nigatu *et al, 2015* | 1 | 1 | 0 | 1 | 1 | 1 | 2 | 7 |
